# Interocular suppression prevents interference in a flanker task

**DOI:** 10.3389/fpsyg.2015.01110

**Published:** 2015-08-11

**Authors:** Qiong Wu, Jonathan T. H. Lo Voi, Thomas Y. Lee, Melissa-Ann Mackie, Yanhong Wu, Jin Fan

**Affiliations:** ^1^Department of Psychology, Peking University, Beijing, China; ^2^The Graduate Center, City University of New York, New York, NY, USA; ^3^Department of Psychology, Queens College, City University of New York, Queens, NY, USA; ^4^Beijing Key Laboratory of Behavior and Mental Health, Peking University, Beijing, China; ^5^Key Laboratory of Machine Perception (Ministry of Education), Peking University, Beijing, China; ^6^Department of Psychiatry, Icahn School of Medicine at Mount Sinai, New York, NY, USA; ^7^Department of Neuroscience, Icahn School of Medicine at Mount Sinai, New York, NY, USA

**Keywords:** executive control of attention, continuous flash suppression, conflict effect, consciousness awareness, flanker task

## Abstract

Executive control of attention refers to processes that detect and resolve conflict among competing thoughts and actions. Despite the high-level nature of this faculty, the role of awareness in executive control of attention is not well understood. In this study, we used interocular suppression to mask the flankers in an arrow flanker task, in which the flankers and the target arrow were presented simultaneously in order to elicit executive control of attention. Participants were unable to detect the flanker arrows or to reliably identify their direction when masked. There was a typical conflict effect (prolonged reaction time and increased error rate under flanker-target incongruent condition compared to congruent condition) when the flanker arrows were unmasked, while the conflict effect was absent when the flanker arrows were masked with interocular suppression. These results suggest that blocking awareness of competing stimuli with interocular suppression prevents the involvement of executive control of attention.

## Introduction

Executive control of attention prioritizes goal-relevant information processing in the presence of competing information or computations (Fan et al., 2002, 2005; Mackie et al., 2013). It has typically been studied by manipulating stimulus and/or response conflict, as in various versions of Stroop tasks (Stroop, 1935), Eriksen flanker tasks (Eriksen and Eriksen, 1974), and Simon tasks (Simon and Berbaum, 1990). In these experiments, executive control of attention is elicited in the presence of conflict. Relative to “congruent” stimuli, these “incongruent” stimuli are usually accompanied by increased response times (RTs) and decreased accuracy. This difference in behavior between incongruent and congruent stimuli defines the conflict effect and is taken as an indication of increased executive control of attention (Botvinick et al., 2001; Fan et al., 2003, 2007, 2008).

An important issue is the relationship between executive control of attention and conscious awareness. Because mechanisms involved in the executive control of attention are typically thought to operate in a top-down manner, classical theories propose it to be exclusive to the domain of conscious cognition (Posner and DiGirolamo, 1998; Dehaene and Naccache, 2001; Jack and Shallice, 2001). This is in contrast with automatic processing, which is assumed to necessarily occur outside of conscious awareness. However, in the context of a body of inconsistent previous findings, whether or not conscious awareness is necessary to elicit executive control of attention is still under debate (Desender and Van den Bussche, 2012; Kiefer, 2012; Kunde et al., 2012; van Gaal et al., 2012; Ansorge et al., 2014; Sterzer et al., 2014).

Unconscious influence of executive control of attention has primarily been investigated with subliminal sequential paradigms where a masked priming stimulus is briefly presented before a target stimulus (e.g., Klotz and Wolff, 1995; Naccache and Dehaene, 2001; Klapp, 2007). Although the prime is masked and thus phenomenally outside of awareness, responses to the target have been shown to be facilitated by congruent primes and hindered by incongruent primes, presumably demonstrating that visual awareness is unnecessary for executive control. Alternatively, this form of conflict can be conceived as automatic activation of motor responses by unconsciously registered primes (stimulus–response mappings), provided that action planning has already occurred (Ansorge et al., 2002, 2014; Ansorge and Neumann, 2005; Kiesel et al., 2007; Kunde et al., 2012). Specifically, humans set up “action triggers” that connect relevant target stimuli with respective motor responses in advance of stimulus presentation (Kunde, 2003). A masked prime that is akin to an action trigger can automatically initiate the corresponding response in the absence of intentional control, even though the prime remains below the threshold of awareness. Thus, the effectiveness of subliminal priming stimuli in sequential paradigms could reflect bottom-up response activation rather than top-down executive control.

In contrast, simultaneous presentation of target and flankers resulting in stimulus and response conflict has been theorized to elicit mechanisms of control in a top-down manner. According to an information theory account of executive control of attention, the conflict effect is attributed to an increase in information uncertainty associated with the target (Fan, 2014; Fan et al., 2014), and is susceptible to top-down influences on ongoing or completed stimulus processing. For example, in flanker tasks, arrows pointing in the same direction can be grouped together. The incongruent condition induces an additional possible response relative to the congruent condition, and thus increases the uncertainty level associated with the central target. This uncertainty increase triggers executive control of attention to resolve the ongoing conflict from the flankers in order to prioritize goal-related target processing. Thus, the effectiveness of competing information to elicit conflict in a simultaneous paradigm reflects intentional detection and resolution of the conflict among responses. It is still unknown, however, whether conscious awareness is required for executive control of attention when conflicting information and target stimulus are presented concurrently.

The most commonly used technique to render stimuli invisible is backward masking, in which the visibility of a very brief stimulus is degraded by the presentation of a succeeding visual pattern (Breitmeyer and Öğmen, 2006). Backward masking is only effective at rendering a priming stimulus invisible for a very brief (i.e., tens of milliseconds) duration (Macknik, 2006), which is much shorter than the duration in typical flanker tasks (at least hundreds of milliseconds) where flankers and the target arrow are presented simultaneously (Eriksen and Eriksen, 1974; Fan et al., 2002). A more powerful technique to interfere with awareness of the visual input is interocular suppression (i.e., continuous flash suppression, CFS), where a temporally dynamic high-contrast image sequence presented to one eye degrades the visibility of a stimulus presented to the other eye (Fang and He, 2005; Tsuchiya and Koch, 2005). Because interocular suppression allows for extended periods (seconds, rather than milliseconds) of invisibility and unawareness of stimuli (Shimaoka and Kaneko, 2011; Stein and Sterzer, 2011), it is an ideal method to investigate the role of conscious awareness in high-level cognitive operations that are assumed to require relatively long processing times (Peremen and Lamy, 2014; Yang et al., 2014).

Additionally, several lines of research have investigated the variability of depth of non-conscious processing with different techniques (Izatt et al., 2014; Peremen and Lamy, 2014). For example, non-conscious processing of a target stimulus could be enhanced by relevant primes that are made invisible because of visual masking (i.e., backward masking) or near-threshold presentation (Naccache and Dehaene, 2001; Naccache et al., 2002). However, visual adaptation effects have been eliminated, or at least substantially reduced, during interocular suppression (Moradi et al., 2005; Blake et al., 2006). These results suggest that interocular suppression possibly interrupts registration of stimuli at an early stage of visual processing, while visual masking would allow partial read-out of information, although without subjective awareness. Although it is evident that conflict information suppressed by visual masking can influence responses to the target (for alternative explanations, see Van den Bussche et al., 2009; Peremen and Lamy, 2014), it still important to know whether this type of executive control of attention elicited by a simultaneous paradigm can occur during interocular suppression.

In this study, we aimed to address this question specifically. We hypothesized that executive control of attention is a top-down conscious process that operates only on information that has reached higher-level processing. If this is true, it implies that awareness of stimuli is necessary to elicit executive control of attention. In two experiments, we used CFS to mask the flanker arrows via two different manipulations of target-flanker eye presentation. We predicted that when masking was absent, we would obtain the conflict effect: incongruent flankers should lead to prolonged RTs and lower accuracy compared to congruent flankers; however, the conflict effect would be absent when masking was present.

## Materials and Methods

### Participants

Thirty individuals participated in Experiment 1, and 31 individuals participated in Experiment 2. The participants were recruited from the Psychology 101 subject pool at Queens College of the City University of New York (CUNY) and given class credit for their participation. All participants had normal or corrected-to-normal vision, and signed informed consent forms prior to the start of the experimental procedure. The experiments were approved by the Institutional Review Board of CUNY and were run in accordance with the provisions of the World Medical Association Declaration of Helsinki.

Participant’s data were excluded from further analysis if they failed to respond to less than 80% of trials, or on the basis of an additional experimental run that probed the effectiveness of the CFS mask (see Procedure below). Based on these exclusion criteria, the final sample consisted of 28 participants in Experiment 1 (10 females, mean age ± SD, 21.43 ± 5.75 years) and 19 participants in Experiment 2 (10 females, mean age ± SD, 21.16 ± 3.70 years).

### Stimuli and Apparatus

**FIGURE 1:**
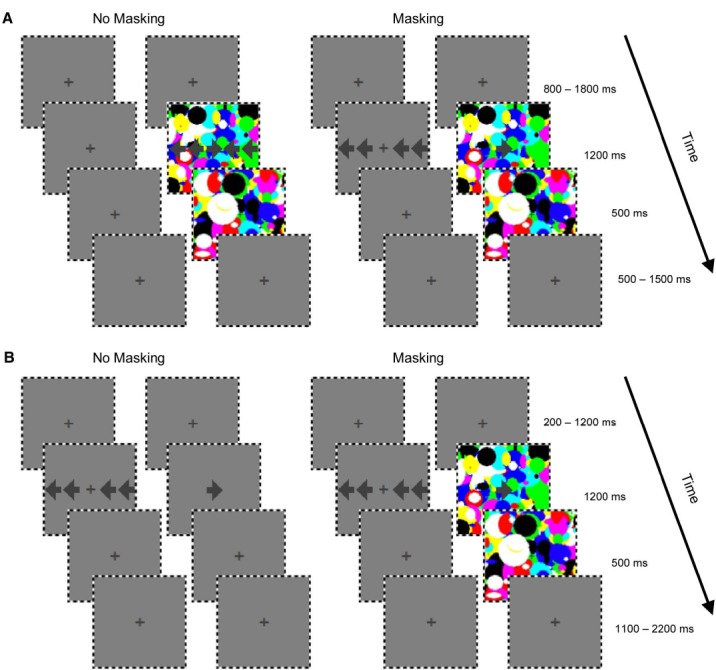
**The sequence of stimuli in each trial from Experiment 1 (A) and Experiment 2 (B).** Each sequence shows an example of the images presented when the target was in the right eye. The left panels (no masking) show conditions where the flanker arrows were not masked, and the right panels (masking) show conditions where the flanker arrows were masked by CFS. **(A)** CFS was present on all trials; by presenting the flanker arrows to the opposite eye from the target arrow and the Mondrians, the CFS masked awareness of the flankers. **(B)** Flanker and target arrows were always presented to different eyes. The presence of the Mondrians in the same eye as the target masked awareness of the flankers. The incongruent condition (with flankers pointing to the opposite direction as the target) is shown in **(A)** and **(B)**. For the congruent condition, the flankers point in the same direction as the target. For the no-flanker condition, there are no flankers displayed.

The stimuli are illustrated in Figure 1. Participants viewed a central *target* arrow (approximately 2.8°) pointing either to the left or right. It could be presented simultaneously with four surrounding *flanker* arrows (approximately 2.8°). The arrows (uncalibrated RGB values = 103) were presented at a low contrast darker than the gray background (uncalibrated RGB values = 128). The target and flanker arrows were presented to the same eye or to different eyes. To facilitate the fusing of the two images, a thin outer square border of alternating black and white bands was presented to both eyes throughout the experiment. In addition, a central fixation cross was presented to both eyes for the duration of the experiment.

In Experiment 1 (Figure 1A), a CFS mask was present on every trial. The mask consisted of a large set of Mondrian images—random colored overlapping ovals that filled the space inside the outer square border (modeled after the CFS masker in Tsuchiya and Koch, 2005). The Mondrians were presented at the rate of 10 Hz. Ovals were used to minimize the number of sharp angles in the mask that could interfere with perceiving the angles that define the arrows. The CFS mask was always presented to the same eye as the target arrow. The flanker and target arrows could be presented to the same eye, or to different eyes. In Experiment 2 (Figure 1B), the CFS mask was presented on 50% of the trials, and was always presented to the same eye as the target arrow. The flanker and target arrows were always presented to different eyes.

A stereoscopic goggle system (ELSA wired 3D goggles with attached head-strap) was used to display the stimuli. The goggle system allowed independent presentation of stimuli to each eye. The screen resolution for each eyepiece was 800 × 600 pixels. Viewing distance to each eyepiece was approximately 2.5 cm. The stimuli were presented using MATLAB (2010b, The MathWorks, Inc.) and routines from the Psychophysics Toolbox Version 3 (Brainard, 1997) on a Mac Pro 5.1 computer (OSX 10.7) with an ATI Radeon HD 5770 GPU. The computer generated visual output at 60 Hz, and each eyepiece received alternating frames of this output at 30 Hz. One eyepiece received the even frames while the other received the odd frames.

### Procedure

In both Experiments 1 and 2, the participants’ task was to indicate whether the target arrow pointed to the left or right by means of a keyboard button press. They were asked to respond as quickly and accurately as possible, and to ignore any other non-target arrows and colorful images. RT and accuracy were recorded by the computer. On 1/3 of the trials, the target arrow was presented alone (*no-flanker condition*), and on the other 2/3 of trials it was presented with the flanker arrows. The direction of the flanker arrows (always the same for all four arrows) was either the same as the direction of the target arrow (*congruent condition*) or the opposite (*incongruent condition*), 1/3 of trials for each.

The visibility of the flanker arrows was manipulated in two different ways. In Experiment 1, the flanker arrows were made invisible (*masking condition*) by presenting them to the opposite eye from the CFS mask and target arrow. The flankers were made visible by presenting them to the same eye as the mask and target (*no-masking condition*). This manipulation is similar to that used in CFS masking experiments (Kang et al., 2011; Yuval-Greenberg and Heeger, 2013) where the visual noise is present on every trial of the experiment. In Experiment 2, the CFS mask was present on 50% of the trials in the same eye as the target. Because the flanker arrows were always presented to the opposite eye from the target arrow, the flanker arrows were invisible when the CFS mask was present. This mask manipulation is similar to the presence of a backward mask in priming experiments (see also Fang and He, 2005), because the visual noise is only present during masking trials.

The six conditions (three flanker conditions × two visibility conditions) of each experiment were randomly presented an equal number of times in each experimental run. The target arrow was randomly presented to the left or right eye with equal probability. Each experiment consisted of four runs, with 96 trials per run. Each run began and ended with a 30 s fixation period, and each trial lasted 4 s, for a total of 7.4 min per run. Participants were given the option of taking a break after each run.

At the start of each trial, a fixation period was presented for a random duration (800–1800 ms in Experiment 1, 200–1200 ms in Experiment 2) in order to jitter the intertrial interval (there was no change on the screen, and only the fixation cross was visible). Then the target arrow, as well as the CFS mask and flanker arrows (if present), appeared for 1200 ms. When the CFS mask was present, it remained on the screen for 500 ms after the offset of the arrows while the fixation cross remained visible in the other eyepiece. On trials where the CFS mask was absent, the fixation cross was seen in both eyepieces for the same duration (500 ms). The continuing presentation of the CFS mask after the target arrow presentation ensured that the flanker arrow’s afterimage did not influence responses. Responses could be recorded at any time up to this point. The fixation cross remained on the screen for the remaining time until the total 4 s trial duration ended.

A fifth run was included to assess whether the flanker arrows were visible despite the presence of the CFS mask. The stimuli in this fifth run were identical to those in the other four runs of the experiment, with the addition of two questions after every trial. Participants were instructed to ignore the target arrow for the fifth run, and to instead watch for the flanker arrows and to respond to the two question prompts. The first question asked whether the flanker arrows pointed to the left or the right. This discrimination question was presented after all trials, whether the flankers were present or not. Participants were told to choose whichever direction “felt right” if no flankers were seen. The second question, presented immediately after the first question, asked whether or not participants saw any flanker arrows. Participants were told to respond “yes” to this one-interval yes/no (Y/N) question if they believed they might have seen the flanker arrows.

In addition to excluding participants who responded to less than 80% of trials, we also excluded participants on the basis of their performance in the fifth run. The exclusion criteria were: (1) responding to less than 80% of questions in the final run, determined by checking the distributions of “percent with responses made” and selecting the cutoff if the distribution was binomial; (2) for the two discrimination and detection questions, *d*′ outside ± 1.0, for trials when the flankers were masked. Note that for calculating *d*′ of the first discrimination question, “right” was arbitrarily defined as signal and “left” as noise. *d*′ is a measure of detection sensitivity in the presence of noise, independent of response bias (Green and Swets, 1966). It has been suggested that with a *d*′ of 0 the individual cannot discriminate between signal and noise, whereas a *d*′ of 1 suggests medium performance and a *d*′ of 4.65 suggests optimal performance (Hortensius et al., 2014). The negative value of *d*′ does not mean no sensitivity, but rather can arise through sampling error or response confusion (responding “yes” when intending respond “no,” and vice versa) (Stanislaw and Todorov, 1999). Given the possible confounding of response confusion, the exclusion criterion of *d*′ < –1 was also made.

## Results

All participants included in the final analysis could not reliably identify the direction of the flankers on the first discrimination question (mean accuracy ± 1 SD = 50% ± 5% in Experiment 1, 50% ± 9% in Experiment 2) when the flankers were masked, but could easily perform this task when the flankers were not masked (98% ± 3%, Experiment 1 and 2). Mean sensitivity (*d*′) on the Y/N question was close to 0 (mean *d*′ ± 1 SD = –0.09 ± 0.37 in Experiment 1, –0.20 ± 0.25 in Experiment 2) for trials when the flankers were masked.

**FIGURE 2:**
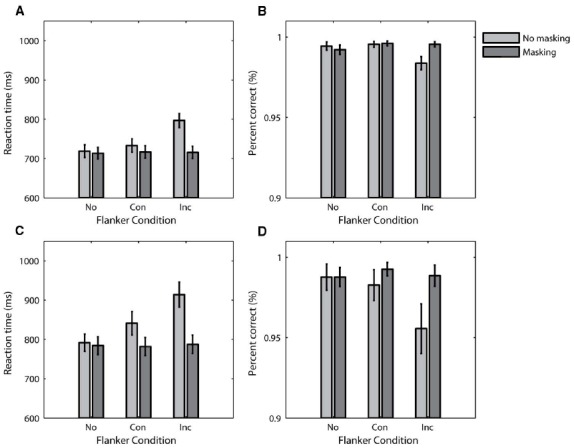
**Behavioral data from Experiment 1 (A,B) and Experiment 2 (C,D).** The mean RT data are plotted as a function of flanker condition in **(A,C)**, and the mean accuracy data is similarly plotted in **(B, D)**. Error bars plot ± 1 standard error of the mean. No, No Flankers; Con, Congruent Flankers; Inc, Incongruent Flankers.

The RT data from Experiment 1 are plotted as a function of flanker condition in Figure 2A. The light gray and dark gray bars represent data from the no-masking and masking conditions, respectively. The main feature of the data is the difference in pattern between the visibility conditions. RT varied as a function of flanker condition for the no-masking condition, but not the masking condition. This was confirmed by a repeated-measures ANOVA with participant as a random effect and flanker and visibility as fixed effects, which indicated a significant flanker by visibility interaction [*F*(2,54) = 82.73, *p* ≤ 0.001]. Simple planned comparisons confirmed that RT varied with flanker condition for the no-masking condition [*F*(1,27) = 127.19, *p* ≤ 0.001], but not the masking condition (*F* < 1). Unsurprisingly, there was no significant difference in RT in the no-flanker conditions [*F*(1,27) = 1.13, *p* = 0.30] because the visual stimuli were the same on these conditions. The mean conflict effect for the no-masking condition was significantly different from 0 [mean RT difference ± 1 SD = 63.50 ms ± 29.80 ms, *t*(27) = 11.28, *p* < 0.001], but not for the masking condition [mean conflict effect ± 1 SD = –1.21 ms ± 20.97 ms, *t*(27) = –0.31, *p* = 0.76].

The accuracy data from Experiment 1 follow a similar pattern, and are plotted as a function of flanker condition in Figure 2B. A repeated-measures ANOVA with participant as a random effect and flanker and visibility as fixed effects indicated a significant flanker by visibility interaction [*F*(2,54) = 4.43, *p* = 0.02]. Simple planned comparisons again confirmed that this interaction is due to an effect of flanker condition on accuracy for the no-masking condition [*F*(1,27) = 9.00, *p* = 0.006] but not the masking condition (*F* < 1). There was no significant difference in accuracy between the no-flanker conditions (*F* < 1). The mean conflict effect for the no-masking condition was again significantly different from 0 [mean accuracy difference ± 1 SD = –1.2% ± 2.1%, *t*(27) = –3.00, *p* = 0.006], but not for the masking condition [mean accuracy difference ± 1 SD = –0.1% ± 1.1%, *t*(27) = –0.27, *p* = 0.79].

The RT and accuracy data from Experiment 2 are plotted in Figures 2C,D. The pattern of both measures is very similar to the data in Experiment 1. For both RT and accuracy, repeated-measures ANOVAs with participant as a random effect and flanker and visibility as fixed effects indicated a significant flanker by visibility interaction [*F*(2,36) = 25.37, *p* ≤ 0.001 for RT; *F*(2,36) = 5.02, *p* = 0.01 for accuracy]. Simple planned comparisons demonstrated an effect of flanker condition when there was no masking [*F*(1,18) = 40.38, *p* ≤ 0.001 for RT; *F*(1,18) = 8.92, *p* = 0.008 for accuracy], but failed to show an effect of flanker condition when there was masking [*F*(1,18) = 1.83, *p* = 0.19 for RT; *F*(1,18) = 2.03, *p* = 0.17 for accuracy]. In addition, we did not find any significant differences between the no-flanker conditions (*F* < 1 for both RT and accuracy). The conflict effects were similar to those in Experiment 1. For both RT and accuracy, the mean conflict effects in the no-masking conditions were significantly different from 0 [mean RT difference ± 1 SD = 72.68 ms ± 49.87 ms, *t*(18) = 6.35, *p* < 0.001; mean accuracy difference ± 1 SD = –2.7% ± 4.0%, *t*(18) = –2.99, *p* = 0.008]. On the other hand, the mean conflict effects in the masking conditions were indistinguishable from 0 [mean RT difference ± 1 SD = 5.67 ms ± 18.30 ms, *t*(18) = 1.35, *p* = 0.19; mean accuracy difference ± 1 SD = –0.4% ± 1.3%, *t*(18) = –1.42, *p* = 0.17].

Across two experiments we showed non-significant conflict effects under the masking condition, which may support our null hypothesis that awareness and executive control of attention are closely related. However, an alternative interpretation of these non-significant results could be that the data are insensitive in distinguishing the theory from the null hypothesis. In fact, a non-significant *p*-value, no matter “how non-significant it is,” does not distinguish evidence for the null from no evidence at all (Royall, 1997). To assess whether the absence of conflict effects under interocular suppression indicated evidence for null or just insensitivity, we calculated Bayes factors (B) to determine the relative strength of evidence for null and alternative hypotheses (Dienes, 2008, 2014). The value of B means that the data are B times more likely under the alternative than under the null hypothesis. The conventional standard for assessing substantial evidence for the null is a value of B less than 1/3, while values between 1/3 and 3 are counted as data insensitivity (Jeffreys, 1998). The Bayes factors were calculated using free online Dienes (2008) Bayes calculator (http://www.lifesci.sussex.ac.uk/home/Zoltan_Dienes/inference/bayes_factor.swf) by specifying a uniform distribution with all population parameter values from the lower to the upper limit equally plausible. The upper limit was indicated by the conflict effect under the no masking condition where the flankers were consciously visible, while the lower limit was set with a default of 0. In Experiment 1, the values of B for both RT and accuracy were smaller than 1/3 (B_*U*[0,64]_ = 0.06 for RT, and B_*U*[0,1.2]_ = 0.28 for accuracy), indicating substantial evidence for the null hypothesis. In Experiment 2, the value of B for RT was less than 1/3 (B_*U*[0,73]_ = 0.33), which also supported the null hypothesis. However, the value of B for accuracy was slightly greater than 1/3 (B_*U*[0,2.71]_ = 0.7), indicating a lack of sensitivity of the accuracy measure. Overall, the results provide strong evidence for the phenomenological absence of unconscious processing.

## Discussion

Across two experiments, we demonstrated the presence of conflict effects when the flankers were unmasked by CFS. When the flanker arrows were masked, they had no significant effect on performance as measured by RT or accuracy. In fact, performance under masked conditions was indistinguishable from conditions when no flankers were presented. These results suggested a close relationship between executive control of attention and conscious awareness. It should be noted that our findings could not lead us conclude a necessary role of awareness in executive control of attention. The absence of conflict effect when the flanker arrows were masked from awareness could be attributed to the deep suppression with CFS-like technique. Specifically, that degraded the representation of the flankers to an extent and thus abolished the measure of executive control of attention.

We confirmed the effect of masking using both an objective identification measure and a subjective detection measure. In the additional fifth run, using stimuli identical to those in the rest of the experiment, participants were asked to judge the direction of the flankers and then subjectively report the presence of the flankers. We showed that participants were unable to identify the direction of flankers or detect their presence under the masking condition. Although such demonstrations of lack of awareness are common in masked priming experiments, some care is warranted in interpreting these kinds of results (Lau, 2008). Perceptual awareness is usually equated with performance in sensitivity measures, particularly in forced-choice discrimination/detection tasks, and unawareness is synonymous with null sensitivity of the stimulus. It has been suggested, however, that task performance provides an inconsistent measure of conscious awareness, which should be compensated for with subjective reports of seeing, guessing or rating the clarity of conscious experience (Lau and Passingham, 2006; Sandberg et al., 2010).

Classical theories propose that executive control of attention is closely linked to conscious cognition (Posner, 1994; Velmans, 1996; O’Regan and Noë, 2001; Mole, 2008; De Brigard and Prinz, 2010). Studies have shown that in a prime-target task, the presence of a conflict effect (between prime and target) on a given trial as a function of conflict in the preceding trial occurs only when the prime is visible but not masked, while there is no evidence of such an effect when the prime is invisible in the preceding trial (Kunde et al., 2003; Ansorge et al., 2011; Desender and Van den Bussche, 2012). These studies suggest that conscious experience of a preceding conflict effect is necessary to evoke executive control for the subsequent trial. These studies aimed to investigate the necessity of awareness for the adaptation effect called the Gratton effect (Gratton et al., 1992). Because the prime is presented earlier than the target, a sensorimotor activation account is usually employed to explain the conflict effect, especially when the prime and target are the same, compared to when primes are different from the target (Fagioli et al., 2007; Ansorge et al., 2011). In the present study, the flanker and target were presented simultaneously, thus avoiding the potential alternative explanation of sensorimotor activation. According to an information theory account of executive control of attention, the conflict could be due to an increase in information uncertainty associated with the target (Fan, 2014). This uncertainty increase triggers executive control of attention to resolve the ongoing conflict from the flankers, in order to prioritize goal-related target processing. Therefore, activation of stimulus–response mappings cannot account for these results. In our experiment, the difference between the masked and unmasked flanker conflict conditions was whether or not there were interocular suppression that masked the flankers out of awareness, consequently ruling out several potential confounds present in previous studies, such as differential time of presentation of masked and unmasked stimuli.

Our results showed that the conflict effect elicited by consciously perceived incongruent flankers was abolished when the flankers were masked out of awareness, consistent with the view that high-level cognitive processes only operate on events of which we are aware (Dehaene and Naccache, 2001, for an overview, see Jack and Shallice, 2001; Hommel, 2007). In addition, it has been previously suggested that attention may modulate processing of invisible stimuli (Naccache et al., 2002; Bussche et al., 2010; Yang et al., 2014), with supporting neuroimaging evidence in CFS-like paradigms (Bahrami et al., 2007). Notably, however, in our study, the flankers (masked or unmasked) were always presented in close proximity to the target, and consequently, the attentional spotlight was directed toward the masked stimuli for the entirety of the masked flanker trials. We found no evidence to support the idea that masked flanker processing was enhanced by attentional focus. These results suggest that visual awareness and executive control of attention are closely related. It should be noted, however, that these results cannot lead us to conclude that consciousness and attention are inseparable. There is evidence showing that conscious awareness and visual attention are supported by distinct neuronal mechanisms (Watanabe et al., 2011; Horga and Maia, 2012). It has also been shown that some high-level operations, under certain conditions, can be deployed independently of visual awareness (Soto and Silvanto, 2014; Jachs et al., 2015). For example, recent research has shown that critical relevant information that goes undetected can permeate to working memory and may enable non-conscious information to be maintained and bias subsequent perceptual processing, and engagement of prefrontal cortex (Soto et al., 2011; Dutta et al., 2014; Pan et al., 2014).

In previous studies using subliminal sequential priming paradigms, the prime is masked either by introducing noise stimuli before and/or after the presentation of the prime, or by manipulating the duration and timing of the prime (Cheesman and Merikle, 1984; Dehaene et al., 1998; Heinemann et al., 2009; Bahrami et al., 2010; Van Opstal et al., 2011). Because the prime is presented with only a relatively short duration in these studies, the priming effect might not reflect intentional top-down processes that are relatively slow to develop (Mulckhuyse and Theeuwes, 2010). Unlike the backward masking technique that is effective only when stimuli are presented very briefly (typically for less than 100 ms), CFS-induced suppression that can last on the order of seconds (Shimaoka and Kaneko, 2011). This makes it a particularly well-suited technique to investigate the relationship between conscious awareness and high-level cognitive processing, which may require a relatively long processing time. There is evidence to suggest that the masking effect of CFS is initially weak, and increases with successive presentations, reaching its maximum effectiveness and plateauing after approximately 500 ms (Tsuchiya et al., 2006; Yang et al., 2014). In our study, the target arrow and flankers were simultaneously presented for 1200 ms, much longer than the typical durations of tens to hundreds of milliseconds used in priming experiments. In addition, this way of presenting stimuli was more similar to those used in traditional executive control studies. Note that the relatively long stimulus duration was not related to whether or how unconscious processing happened. For example, it has been shown in priming paradigms that primes with shorter durations are more effective in eliciting unconscious processing (Barbot and Kouider, 2012).

A more likely explanation of our results could be that the complete lack of a conflict effect under the masking condition is due to a deeper suppression of stimuli by CFS compared to masked priming paradigms (Peremen and Lamy, 2014). It has been argued that non-conscious influences of primes with short presentation duration or backward masking may be attributed to partial conscious perception (Kouider and Dehaene, 2007). In contrast, CFS is a more effective masker that is assumed to have its effects at an early stage and thus impede further high-level processes (Yuval-Greenberg and Heeger, 2013). For example, a number of studies have failed to obtain evidence for unconscious processing of high-level information rendered invisible with CFS (Moradi et al., 2005; Blake et al., 2006; Stein and Sterzer, 2011). In our study, the absence of conflict effects during CFS in our experiments is in line with previous findings that high-level visual unconscious processing is comparably limited under interocular suppression (Tong et al., 2006; Almeida et al., 2008; Lin and He, 2009; Stein and Sterzer, 2014). It should be noted, however, the deep suppression under CFS might be at risk of being too deep, consequently leading to false-negative findings (Sterzer et al., 2014). In fact, our results showed that performance under masked conditions was indistinguishable from the condition in which no flankers were presented, suggesting that CFS substantially reduced the perceptual representation of flankers and abolished the measure of executive control of attention. In addition, the effectiveness of CFS masking was confirmed with an additional procedure in which performance on the discrimination/detection of flankers was assessed. This measure might be too strict given that the strength of suppression varies during CFS. For example, the stimulus contrast should be set low enough to ensure that it would not break CFS for the duration of the experiment. Thus, the absence of unconscious processing could result from the use of the interocular suppression technique of CFS, rather than abolishment of awareness. This concern could possibly be addressed by collecting subjective measures of awareness on a trial-by-trial basis, while comparing performance on trials when the stimuli are fully suppressed versus partially suppressed (Stein and Sterzer, 2011; Stein et al., 2012).

Neuroimaging studies using backward masking paradigms showed that activity in regions associated with executive control appears to be attenuated when the incongruent prime stimulus is masked. For example, one study showed activity in the anterior cingulate cortex (ACC) during a priming task involving a categorical determination of numbers greater or less than five (Dehaene et al., 2003). Although there was a non-zero behavioral effect size when the prime was both masked and unmasked, ACC activation related to prime-target conflict was present only when the prime was unmasked. The behavioral pattern of our results resembles this pattern of neural activity, and provides converging evidence supporting the necessity of awareness of the stimuli in executive control of attention. However, other studies found ACC activation with unconscious conflict (Ursu et al., 2009), and inferior frontal cortex (IFC) and pre-supplementary motor area (pre-SMA) activation with backward masked no-go stimuli (van Gaal et al., 2010). Further studies using reliable masking techniques are required to clarify the neural bases of unconscious stimuli processing.

Continuous flash suppression and backward masking may fundamentally rely on different mechanisms of interference. The effects of primes masked by both CFS and backward masking have been shown to differ: unconscious priming effects are restricted to a specific category of primes rendered invisible with CFS, whereas the priming effects can be obtained across a range of different categories of primes rendered invisible through backward masking (Almeida et al., 2008). This finding has led to the speculation that CFS and backward masking might interfere with information passing through different neural pathways (Almeida et al., 2013). CFS has been shown to interfere with activity in early visual cortex (Yuval-Greenberg and Heeger, 2013), and backward masking interferes with activity in the superior colliculus and pulvinar (Dehaene et al., 2001). The lowest contrast target rendered invisible by CFS evoked V1 activity that was statistically indistinguishable from the mask-only (no target) condition (Yuval-Greenberg and Heeger, 2013). This interference is maintained through higher regions in the visual pathway, (Fang and He, 2005; Tsuchiya and Koch, 2005; Jiang et al., 2006; Watanabe et al., 2011). By using CFS, we can attribute the lack of awareness in our masking conditions to the obliteration of the information of flanker arrows before passing through V1. Although CFS seems to be an elegant way to suppress a stimulus without changing its physical properties and has gained increasing popularity for studying visual awareness (Fang and He, 2005; Tsuchiya and Koch, 2005; Jiang et al., 2006; Watanabe et al., 2011), some care is warranted in using this technique. CFS is based on affecting the gain of neural responses in early visual cortex, which is akin to reducing stimulus contrast. By changing the context in which the stimulus is presented, CFS would degrade the representation of the masked stimulus and suppress the stimulus more deeply compared to other visual masking paradigms. An interesting possibility is to perform the present experiments using a different form of masking, one that selectively interferes with only regions further up in the visual processing stream. In a technique referred as “chromatic flicker fusion” (CFF), two isoluminant and opposing colored stimuli are simultaneously presented to both eyes and flicker dramatically in counter-phase with each other at a temporal frequency above the flicker fusion threshold (∼30 Hz) (Hoshiyama et al., 2006). Although CFS and CFF can render stimuli subjectively invisible with supposedly comparable effectiveness, unconscious information that never leaves the occipital lobe using CFS is decodable within temporal and frontal regions using CFF (Fogelson et al., 2014). Thus, CFF may be a more sensitive technique for measuring unconscious high-level processing than CFS.

In considering the possibility of an effect of unconscious stimuli on executive control of attention, our study has not addressed whether invisible emotional or threatening stimuli can elicit executive control of attention. There is reason to suspect that if executive control of attention would act on unconscious stimuli, it would do so for stimuli that have the greatest behavioral and adaptive relevance (van Gaal et al., 2010; Ansorge et al., 2014). The flexibility of top-down executive control of attention, for example, notably includes the ability to shift attention between goal-relevant stimuli and other stimuli in the environment that grab attention in a bottom-up fashion (Fan, 2014). There is some evidence to suggest that information from suppressed facial stimuli is indeed processed (Vuilleumier et al., 2001; Pasley et al., 2004; Vuilleumier, 2005; Finkbeiner and Palermo, 2009; Smith, 2012), as well as information from stimuli that are temporally surprising (McCormick, 1997; Mulckhuyse et al., 2007). While the so-called “refined” theories of automaticity attribute flexibility to unconscious processes as well (Kiefer, 2012), much further work is required in this area in order to arrive at a viable conclusion.

We conclude from this study that blocking awareness of competing stimuli using interocular suppression prevents conflict processing. Careful examination and a deeper understanding of the mechanisms involved in masking stimuli from awareness is necessary in order to fully understand the relationship between executive control of attention and awareness.

## Author Contributions

QW and JLV contributed equally to experimental design, data collection, and drafting of the work. TL contributed to data analysis, interpretation of data, and drafting of the work. MM contributed to the interpretation of data and drafting of the work. YW contributed to the interpretation of data and drafting of the work. JF was involved in all of these aspects. All authors approved the final version to be published and agreed to be accountable for all aspects of the work in ensuring that questions related to the accuracy or integrity of any part of the work are appropriately investigated and resolved.

### Conflict of Interest Statement

The authors declare that the research was conducted in the absence of any commercial or financial relationships that could be construed as a potential conflict of interest.
